# Soda Taxes: The Importance of Analysing Policy Processes: Comment on "The Untapped Power of Soda Taxes: Incentivising Consumers, Generating Revenue, and Altering Corporate Behaviours"

**DOI:** 10.15171/ijhpm.2017.126

**Published:** 2017-10-21

**Authors:** Yann Le Bodo, Philippe De Wals

**Affiliations:** ^1^Evaluation Platform on Obesity Prevention (EPOP), Quebec Heart and Lung University Institute Research Center – Laval University (Université Laval), Quebec City, QC, Canada.; ^2^Faculty of Nursing, Laval University (Université Laval), Quebec City, QC, Canada.; ^3^Department of Social and Preventive Medicine, Faculty of Medicine, Laval University (Université Laval), Quebec City, QC, Canada.

**Keywords:** Soda Tax, Sugar, Food and Nutrition, Policy Process, Health Promotion

## Abstract

Sarah A. Roache and Lawrence O. Gostin’s recent editorial comprehensively presents soda taxation rationales from a public health perspective. While we essentially agree that soda taxes are gaining momentum, this commentary expands upon the need for a better understanding of the policy processes underlying their development and implementation. Indeed, the umbrella concept of soda taxation actually covers a diversity of objectives and mechanisms, which may not only condition the feasibility and acceptability of a proposal, but also alter its impact. We briefly highlight some conditions that may have influenced soda tax policy processes and why further theory-driven case studies may be instructive.

## Introduction

Sarah A. Roache and Lawrence O. Gostin’s recent editorial highlights various soda taxation logics from a public health standpoint, including a price-induced consumption disincentive, the generation of revenues to be earmarked for health promotion initiatives, and the stimulation of product reformulation by the industry.^1^ As it has been argued elsewhere,^2^ we essentially agree with the authors about the importance to tackle sugar-sweetened beverage consumption and the fact that soda taxation could be part of a portfolio of nutrition-enhancing policies aimed at preventing chronic diseases. We also agree about the importance to carefully consider potential concerns, eg, increasing socioeconomic inequalities, harmful substitution, and appropriate use of revenues. Despite promising results in several jurisdictions (eg, Berkeley [CA] and Mexico),^3,4^ the editorial righty calls for evaluation efforts in order to assess the impact of soda taxes.^1^ In this commentary, we would like to expand this imperative upon the need for a better understanding of the policy processes underlying the development and implementation of such policies.

## Behind the Scenes, the Importance of Policy Processes

Indeed, despite the “global momentum for soda taxes” described by Roache and Gostin,^1^ this type of intervention remains controversial.^5^ While soda taxes have been enacted or announced in more than 20 jurisdictions across the world,^2^ debates keep going in many others eg, in Australia,^6^ New Zealand,^6^ Canada,^7^ Columbia^8^ or the Philippines.^9^ Therefore, understanding what conditions favor or preclude the elaboration of soda taxes remains of interest. Furthermore, the umbrella concept of soda taxation can actually take many shapes, be this in terms of tax objectives (focused on consumption, revenue generation and/or incentive for reformulation), tax type (eg, excise or at the point of purchase), tax rate (from 2% to more than 30%), tax scope (eg, including or not non-caloric sweetened beverages), tax base (eg, flat rate or indexed on the sugar content), tax scale (eg, local, state or national) and use of tax revenue (eg, earmarked or not).^2,10^ These considerations and parameters may not only condition the feasibility and acceptability of a soda tax proposal, but it may also influence its impact. This makes the analysis of soda tax policy processes all the more relevant. For example, considering that the forthcoming UK soda tax indexed on sugar content has already encouraged manufacturers to reformulate beverages (which was the primary objective stated by the government),^11^ Roache and Gostin rightly highlight that understanding the circumstances having led to such a tax could be valuable for other jurisdictions. They underline key conditions that may have facilitated the policy process, including the step-by-step approach adopted by the government and the flexibility of the graduated tax scheme for manufacturers.^1^ In sum, although less efforts have been dedicated to study soda tax policy processes than soda tax potential and actual impacts,^2^ research on the former can complement research on the latter, as further illustrated in the next section.

## Conditions Influencing the Prospect for Health-Related Food Taxes

Some publications, heterogeneous in purpose, methods and format, bring insights into the conditions that may have favored the adoption of health-related food taxes. To briefly name a few, the high prevalence of obesity and non-communicable diseases as well as the recognition of pervasive consumption of sugary, salty and fatty foods in the population may be a precondition to put a tax on the agenda, as described in Mexico^12^ and Pacific countries.^13^ Additionally, the prospect for health-related food taxes often appears to be related to budgetary considerations, not least because finance authorities generally administer such policies. Therefore, the degree of cooperation between public health and finance policy-makers may actually be critical to make soda taxation feasible and palatable, as shown in Pacific countries,^14^ Mexico,^12^ Barbados,^15^ Colorado,^16^ Philadelphia^17^ or the Cook County in Illinois.^18^ In these last two cases, public health motives were even minimized in order to highlight the budgetary rationale of the tax, whose revenue was earmarked respectively for education and public employment purposes. A budgetary rationale has also predominated in France in 2011^19^ and in Belgium in 2015,^20^ where low excise (less than 0.1 €/litre) and large-scope soda taxes (including non-caloric sweetened beverages) have been adopted as part of large tax reforms. This may have facilitated tax enactment in the short-term, but effects on behaviours may be questioned since higher tax rates focused on caloric sweetened drinks are generally recommended to reduce sugar consumption.^21^ In contrast, if the tax explicitly aims to raise soda prices and curb consumption, then pro-taxation advocacy efforts financially supported by philanthropic organisations may be essential to face resistance among the population, politicians and other stakeholders. Such efforts likely contributed to the adoption of a tax in Mexico (2013), the city of Berkeley (2014), and several other US jurisdictions (2016).^18,22^ The analysis and dissemination of evidence by public health experts and scientific organisations may also help and stimulate the debate.^12,17^ Without sufficient mobilisation to counter opposition, the prospect may be reduced, as described in the Californian city of Richmond (2012)^18^ or in Hawai’i.^23^ Finally yet importantly, whatever the tax justification, political leadership may be decisive, eg, in the way that Mexican senators have supported the price-oriented tax proposals along the legislative processes in 2012-2013,^12^ in the way the UK chancellor has championed the reformulation-oriented “sugar tax” in 2016,^24^ or in the way Philadelphia’s mayor has advocated for the adoption of a revenue-oriented soda tax by the city council in 2015-2016.^17,18^

At the opposite, several factors may contribute to impair the prospect of health-related food taxes. As Roache and Gostin mention, the industry is generally opposed to tax proposals.^1^ With important resources and various means (eg, communication campaigns, advocacy by front groups, lobbying), manufacturers often denounce eg, the risk that such taxes may bring on the local economy and jobs, their discriminatory nature when focused on particular products, their ineffectiveness to address public health issues (obesity in particular), their regressive nature, the threat they bring about consumer autonomy, or the administrative burden they may generate. Such opposition has been reported eg, in Denmark,^25^ in the United States at the federal level,^18^ the state level (eg, in New York,^26^ Hawai’i^23^) or the local level (eg, in several Californian cities^18,22^), in Mexico^12^ or in South Africa.^27^ It has also been reported in the United Kingdom,^24^ although the graduated tax scheme finally adopted may not be worse for the beverage industry than a flat rate targeting equally all sugary drinks, since it may actually further stimulate reformulation efforts undertaken to meet consumer expectations.^28,29^ Additionally, the lack of political will, majority or consensus to support a tax proposal may be a barrier to the emergence of a tax on the political agenda, its formulation or its adoption. For example, it appears to have been the case in Australia,^6^ Canada,^7^ Colombia,^8^ Luxembourg^30^ as well as in several US states (eg, in Kansas^16^ and Hawai’i^23^). Multiple factors may be related to such unfavourable context, including political reluctance and opposition, adverse economic circumstances, lobbying, lack of local evidence, untimely legislative calendar, legal and administrative constraints, etc.^13,16,21,23,25^ Such factors are frequently covered in the media, but disentangling their respective contribution to the policy process is not obvious.^16^ Finally, the uncertain acceptance of soda taxes among the population may preclude their adoption, especially where it is subject to a local ballot.^18^ Indeed, surveys tend to indicate that health-related food taxes are not popular in comparison to other nutrition policies.^2^ Earmarking tax proceeds to health promotion or social programmes targeting the most vulnerable population groups may boost favourable opinions, but may also go with political, legal or administrative constraints.^12,14,15,18^

Therefore, research on soda tax policy processes can unveil a myriad of influences depending on circumstances, ideas and interests. This is congruent with the lessons of a recent literature review on nutrition policy change.^31^ Nonetheless, over time, identifying critical factors among others may be complex and “elusive.”^32^ This is an area where research at the crossroads of public health and political science can be fruitful.^33^

## Benefit of Theory-Driven Research on Soda Tax Policy Processes

The use of political science theories to better understand health promotion issues and influence policy change appears promising.^32^ Any theory remains refutable, but as Breton and de Leeuw put it,^34^ theories of the policy process “(…) formulate propositions on the conditions under which certain policy phenomena (eg, preferences for certain types of interventions, decisions on implementation issues, allocation of resources, inclusion or exclusion of certain stakeholders, etc) are observed and have an impact on policy outcomes.” In other words, beyond common sense and intuition, appropriate theoretical notions can provide the researcher some keys to understand successful or unsuccessful stories. In the aforementioned literature, theory-driven research on soda taxation policy processes remains scarce but instructive cases exist. For example, Thow and colleagues’ case studies in the Pacific^14^ refer to Sabatier’s advocacy coalition framework (ACF) to analyse how external events (eg, alarming chronic disease prevalence, budgetary shortcomings), well-established tax schemes as well as the interplay of health, finance and other authorities have jointly contributed to justify soft drink taxes despite industry opposition and structural constraints (eg, trade agreements). As a well-known and largely used theory, it is noteworthy that the ACF also provides insightful analyses of tobacco tax policy change.^35^ Another example of theory-driven research is Mosier’s multiple case study on soda taxation proposals in Kansas and Colorado in 2009-2010.^16^ It illuminates the propensity of Kingdon’s multiple streams theory (MST) to explain how the random conjunction of a severe problem (ie, chronic public deficit), available solutions (ie, a package of measures including a revenue-oriented soda tax based on existing mechanisms) and favourable political circumstances (ie, sufficient consensus about a policy already proposed in the past) can lead policy entrepreneurs to take advantage of a policy window to put a soda tax on the agenda and make it adopted. Alongside the ACF and other theories, the MST has been particularly used and recommended to further document obesity prevention policy processes.^36^

## Conclusion

While the “momentum” described by Roache and Gostin^1^ is growing in favor of soda taxation across the world, policy studies grounded in a theoretical framework of social change may contribute to further illuminate what conditions favor the relevancy of a soda tax proposal, its feasibility, its acceptability as well as its proper implementation and evaluation in the long term.

## Acknowledgements

Evaluation Platform on Obesity Prevention (EPOP)’s activities are funded by Laval University, Quebec City, QC, Canada thanks to a development grant from the Fondation-Lucie-et-André-Chagnon. The authors are grateful to the anonymous reviewers for their helpful comments and suggestions on an earlier version of this commentary.

## Ethical issues

Not applicable.

## Competing interests

Authors declare that they have no competing interests.

## Authors’ contributions

Both authors contributed to the conceptualization of the commentary. YLB wrote the first draft of the manuscript, which was commented and edited up to the final version by both authors.

## Authors’ affiliations

^1^Evaluation Platform on Obesity Prevention (EPOP), Quebec Heart and Lung University Institute Research Center – Laval University (Université Laval), Quebec City, QC, Canada. ^2^Faculty of Nursing, Laval University (Université Laval), Quebec City, QC, Canada. ^3^Department of Social and Preventive Medicine, Faculty of Medicine, Laval University (Université Laval), Quebec City, QC, Canada.
